# Reverse-Offset Printing of Polymer Resist Ink for Micrometer-Level Patterning of Metal and Metal-Oxide Layers

**DOI:** 10.1021/acsami.1c08126

**Published:** 2021-08-25

**Authors:** Asko Sneck, Henri Ailas, Feng Gao, Jaakko Leppäniemi

**Affiliations:** VTT Technical Research Centre of Finland, Ltd., Tietotie 3, Espoo FI-02150, Finland

**Keywords:** high-resolution printing, metal mesh electrode, metal patterning, reverse-offset printing, thin-film transistor, transparent conductor

## Abstract

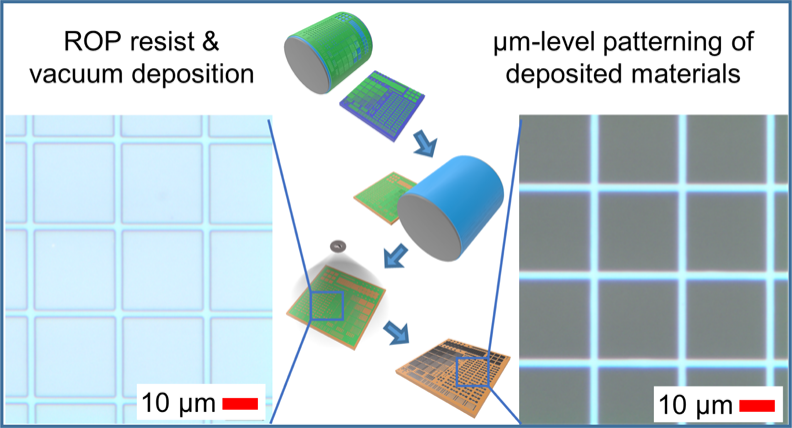

Printed electronics has advanced during the recent decades in applications such as organic photovoltaic cells and biosensors. However, the main limiting factors preventing the more widespread use of printing in flexible electronics manufacturing are (i) the poor attainable linewidths *via* conventional printing methods (≫10 μm), (ii) the limited availability of printable materials (*e.g.*, low work function metals), and (iii) the inferior performance of many printed materials when compared to vacuum-processed materials (*e.g.*, printed vs sputtered ITO). Here, we report a printing-based, low-temperature, low-cost, and scalable patterning method that can be used to fabricate high-resolution, high-performance patterned layers with linewidths down to ∼1 μm from various materials. The method is based on sequential steps of reverse-offset printing (ROP) of a sacrificial polymer resist, vacuum deposition, and lift-off. The sharp vertical sidewalls of the ROP resist layer allow the patterning of evaporated metals (Al) and dielectrics (SiO) as well as sputtered conductive oxides (ITO), where the list is expandable also to other vacuum-deposited materials. The resulting patterned layers have sharp sidewalls, low line-edge roughness, and uniform thickness and are free from imperfections such as edge ears occurring with other printed lift-off methods. The applicability of the method is demonstrated with highly conductive Al (∼5 × 10^–8^ Ωm resistivity) utilized as transparent metal mesh conductors with ∼35 Ω_□_ at 85% transparent area percentage and source/drain electrodes for solution-processed metal-oxide (In_2_O_3_) thin-film transistors with ∼1 cm^2^/(Vs) mobility. Moreover, the method is expected to be compatible with other printing methods and applicable in other flexible electronics applications, such as biosensors, resistive random access memories, touch screens, displays, photonics, and metamaterials, where the selection of current printable materials falls short.

## Introduction

Novel printing methods such as micro-contact printing (μ-CP),^1^ high-resolution gravure,^2^ adhesion contrast planography,^3^ and reverse-offset printing (ROP)^4^ have advanced the minimum attainable line resolution toward micrometer-level printing, hence well beyond that available with conventional printing methods such as gravure, flexography, and inkjet printing (>30 μm). From these high-resolution printing methods, ROP in particular shows great promise for the fabrication of electronic components and circuits as it can deliver high-quality patterns with a submicrometer printing resolution,^5^ micrometer-level overlay printing accuracy,^6^ uniform layer thickness, and rectangular cross section with steep sidewalls, thus producing features resembling those obtained with photolithography.^4^ However, there is a fundamental dearth in the availability of electronic materials as printable inks, which limits the more universal utilization of printing methods in electronics manufacturing. For example, many low work function metals such as Al and Ti cannot be formed as stable, printable nanoparticle (NP) solutions due to their rapid oxidation in air.^7,8^ In addition, the performance of printed oxide materials such as Sn-doped In_2_O_3_ (ITO) is either severely deteriorated from their vacuum-processed counterparts or requires high-temperature processing (>250 °C) to sinter or anneal the inks after the deposition to reach reasonable electrical performance.^9^ Moreover, the cost of printed conductors using Ag or Au NP inks that are commercially available at production-level quantities is dominating in device-level material cost calculations.^8^ Therefore, the use of cheaper metals such as Al would be highly beneficial in cost-conscious applications.

Several patterning methods of vacuum-deposited layers have been developed to address the aforementioned issue regarding limited availability of printable materials. Those include methods originating from a combination of the conventional printing and microfabrication methods, such as printed lift-off^10^ and printed etching,^11^ and stamping methods, such as nanoimprint lithography (NIL),^12^ nanotransfer printing (NTP),^13^ and chemical lift-off lithography (CLL).^14^ Unfortunately, printed lift-off and printed etching using gel-based etchants suffer from “edge ears” of the patterned deposited material that can protrude through subsequent layers. When a sacrificial resist layer is printed using the conventional printing methods, such as inkjet, screen, gravure, or flexographic printing, the liquid ink is prone to interactions with the substrate surface, and as a result, the patterned resist layer will have limited linewidth resolution, considerable line edge roughness, and oblique sidewalls. As the vacuum-deposited material is removed during the lift-off step, the thin layer of the material deposited on the oblique sidewalls will lead to the formation of the edge ears. Similarly, in the case of patterning blanket-deposited layers by printing of etching gel, the edge ears are formed from the residual material that has only partially reacted with the etching gel. In addition, rough and porous edges arise from the uneven spreading of the printed etchant on the vacuum-deposited material, which will ultimately limit the attainable linewidth resolution.

NIL has been developed since the mid-90s as a high-throughput, parallel patterning method that can deliver high-resolution submicrometer patterns without the need of photo- or e-beam lithography steps.^12,15^ In NIL, a stamp fabricated in Si wafer or quartz glass is used to emboss a polymer resist layer on a substrate. This deformation can be molded in the polymer either by applying heating or by UV exposure to harden the resist. The imprinting mold will typically have tapered sidewalls to prevent defects arising from the resist sticking to the mold during a demolding step, which leads to oblique sidewalls in the resulting imprinted resist.^15^ Typically, the patterned resist layer is used as an etching mask for multi-step etching, where in the first step, dry etching is used to remove the residual resist at the bottom of the imprinted pattern, in the second etching step, the deposited layers beneath the resist are patterned, and finally, the resist mask is removed. This requires a careful processing parameter control to avoid unwanted residual resists. Another way to use NIL in patterning of the deposited layers is to use lift-off, where either bi-layer resists^16^ or multi-step processes such as resist profile inversion^17^ or reverse pattern duplication *via* two-step lift-off^18^ are required to avoid the formation of edge ears due to the tapered edges of the imprinted resist.

In nTP, metal layers deposited onto a patterned polydimethylsiloxane (PDMS) stamp can be transferred to a receiving substrate with the help of self-assembled monolayers (SAM)^13^ or adhesive layers^19^ on the substrate to provide strong adhesion to the deposited metal layer. Alternatively, low-surface energy release layers such as fluoropolymers can be used on the stamp to promote the transfer to the receiving substrate.^20^ The method is, however, limited to metals, and in case an adhesive layer is used on the substrate, this layer remains underneath the transferred material, which can be detrimental in some applications such as top contacts to devices.

A patterning method based on the patterning of SAMs, CLL, has been developed originally to pattern vacuum-deposited Au layers.^14^ A patterned PDMS stamp is used to selectively transfer SAMs from a receiving substrate having a SAM layer on top of a previously deposited Au layer. The SAMs attach to the PDMS stamp in the contact areas, thus in an inverse fashion when compared to the SAM printing in μ-CP. The remaining patterned SAM layer is then used as a mask in wet etching of the underlying Au layer. Although the method has recently been expanded to enable patterning of various metals and Ge,^21^ the main challenge with CLL lies in the throughput as the necessary contact time between PMDS and the SAM layer can range from 1 h up to 24 h.^14,21^

In this paper, we leverage the potential of ROP in producing polymer patterns at the micrometer level^22^ and describe the printing of sacrificial polymer resist patterns for a hybrid, scalable, low-temperature, and high-resolution patterning process that enables micrometer-level patterns of various high-quality, vacuum-deposited materials, demonstrated here with evaporated Al and SiO as well as sputtered ITO without any lithography steps. The patterned layers have uniform thickness, sharp vertical sidewalls, and low edge roughness, thus resembling those obtained with conventional photolithography. The patterning method is based on the sequential steps of ROP of a sacrificial polymer resist pattern with sharp vertical sidewalls, vacuum deposition, and lift-off in an organic solvent in ultrasonic bath. The resistivity of the patterned ∼40 nm-thick Al-lines ∼5 × 10^–8^ Ωm (∼2× bulk Al and ∼3.4× bulk Ag resistivity) is similar to resistivity that can be reached at best by using printed Ag NPs *via* alternative sintering methods that are less scalable than thermal sintering.^23^ Notably, this conductivity is obtained at a fraction of the material cost of the printed Ag NPs. The use of the method in applications is demonstrated with transparent conductors based on Al metal meshes and Al source/drain (S/D) electrodes for solution-processed metal-oxide thin-film transistors (TFTs). Importantly, the material palette of printed and flexible electronics can be expanded by the method to materials that cannot be printed while maintaining full compatibility with various printing methods. Therefore, we expect the method to be widely applicable in various flexible electronics applications as it bridges the gap between the materials provided by the current printed electronics and the material demands of the electronics industry.

## Results and Discussion

**Figure 1:**
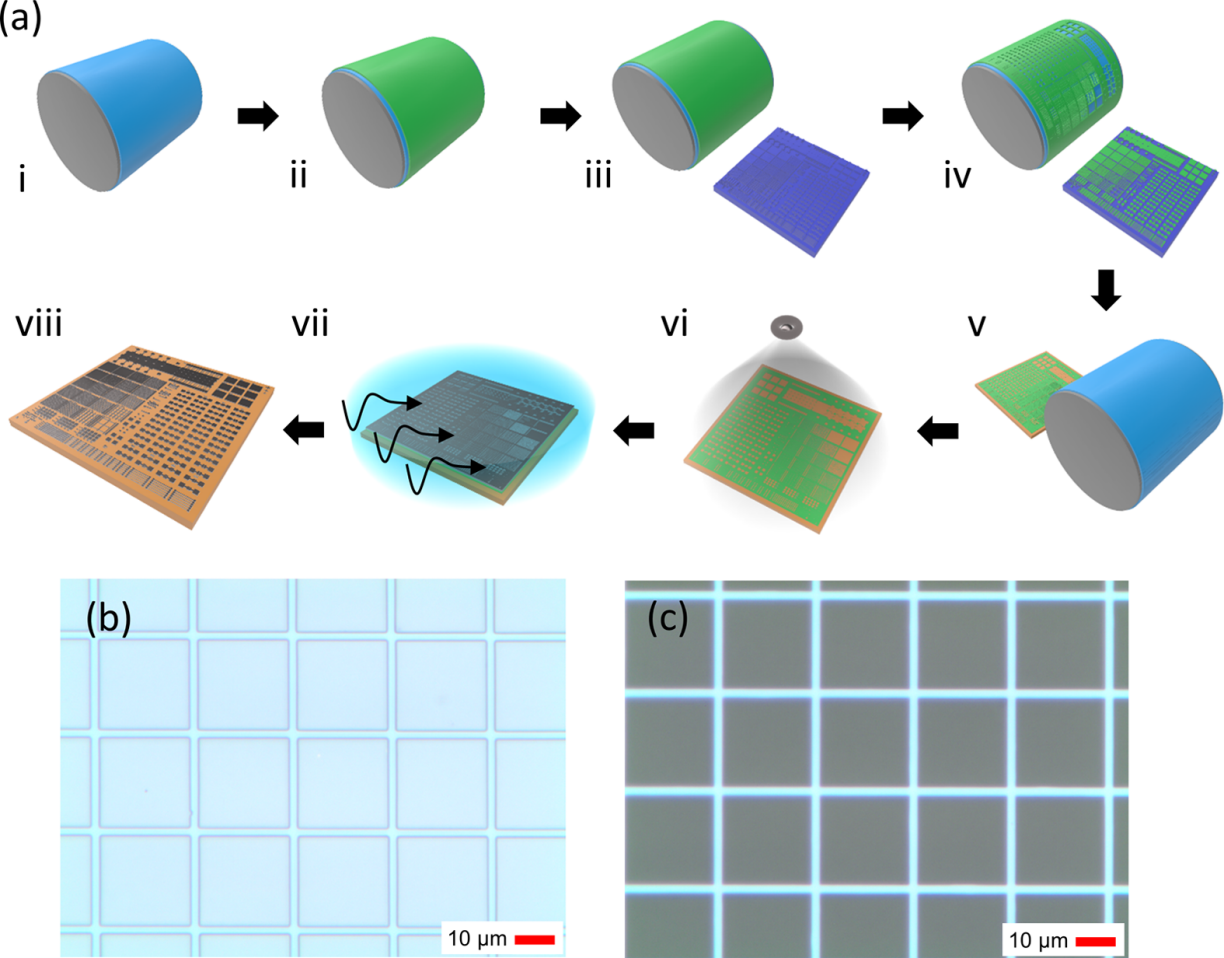
(a) Process schematic of the patterning method: (i) PDMS blanket, (ii) polymer ink coating, (iii) high-resolution cliché, (iv) ink patterning on PDMS, (v) ink transfer from PDMS to the substrate, (vi) patterned polymer ink as the deposition mask, (vii) lift-off in an organic solvent with ultrasonic agitation, and (viii) the final high-resolution pattern on the substrate. (b) Optical microscopy image of the printed polymer resist layer before lift-off with 2 μm gaps. (c) Optical microscopy image of the ITO pattern after lift-off with a 2 μm linewidth.

A schematic image of the hybrid high-resolution patterning method is shown in Figure 1a. The requirements for the polymer ink for the hybrid patterning method are as follows. First, the polymer needs to be soluble in a suitable solvent that allows good wetting on a polydimethylsiloxane (PDMS) blanket surface and forms a semi-dry ink condition on the PDMS through partial evaporation and by partial absorption of the solvent to the PDMS. The semi-dry ink needs to enable patterning at high resolution with sharp, vertical sidewalls and undergo perfect transfer to the printing plate (cliché) from the PDMS. The remaining ink transferred to the substrate needs to form a thick enough layer when completely dried and withstand the thermal impact of the material deposition process (*e.g.*, the heat of condensation and infrared radiation from the molten source in the case of vacuum evaporation) without cross-linking, flowing, or outgassing. Finally, the polymer ink needs to be removable with a solvent (lift-off) and leave no residue after washing.

Three polymer candidates with different polar side groups that could meet the abovementioned criteria were selected for the first ROP printing tests, namely, poly(methyl methacrylate) (PMMA), polyvinylpyrrolidone (PVP), and poly(4-vinylphenol) (PVPh). The polarity of the polymer could assist in obtaining a good adhesion to the cliché to enable perfect transfer.^24^ PMMA has been used widely as an electron-sensitive resist in electron beam lithography for the lift-off process.^25^ PVP has been employed as an additive in photoresists^26^ and printed using μ-CP.^27^ PVPh has been used as cross-linkable gate dielectric for organic thin-film transistors (TFTs)^28^ and also printed using ROP.^22^ The three polymers were dissolved at various weight loadings in various solvents (1-butanol, 2-methoxyethanol, cyclohexanone, ethyl acetate, ethyl lactate, and toluene) that were considered suitable for ROP in terms of their physical properties and absorption to PDMS (Table S1).^4^ In the initial printing tests using the inks, PVP dissolved in 1-butanol and PVPh dissolved either in ethyl acetate or ethyl lactate produced good patterns, whereas PMMA failed to produce patterns (Table S2 and Figure S1). The result of the patterning tests for evaporated Al films is shown after lift-off for 3 wt % PVPh in ethyl lactate and 5 wt % in 1-butanol in Figure S1g and Figure S1h, respectively. The three most promising polymer inks derived from the results of the ink screening tests are listed in Table S3 along with their measured physical parameters.

In the hybrid patterning process, first, a polydimethylsiloxane (PDMS) blanket is coated with a polymer ink, for example, 4 wt % PVPh in ethyl acetate or ethyl lactate, which is used throughout the rest of the work. A short (6 s) oxygen plasma treatment allows the total surface energy of the PDMS blanket to be increased to a sufficient level (from ∼13 to ∼73 mN/m) to promote good wetting of the inks with <30 mN/m surface tension (Figure S2 and Table S4). This helps in achieving a uniform coating on the PDMS, which is important in achieving good patterning. The ink is allowed to reach semi-dry conditions on the PDMS, where the selection of the solvent volatility and absorption to PDMS can be used to tailor the process window of the semi-dry ink state. Then, a high-resolution cliché with desired positive image patterns etched about 5 μm deep into a Si wafer (Figure S3) is brought to contact and pressed against the semi-dry ink. In this work, a hand transfer process is used where a rubber hand roller is used to press the PDMS onto the cliché. The semi-dry ink is patterned on the PDMS by the removal of the ink to the raised area of the cliché. The patterned ink having the negative image of the desired pattern is then transferred (here *via* hand transfer) from the PDMS blanket to the receiving substrate that can be rigid or flexible. The patterned polymer ink on the substrate is dried at low temperature (100 °C) and used as a mask in vacuum deposition. Finally, the polymer ink is dissolved in an organic solvent (*e.g.*, methanol) to give the final high-resolution pattern on the substrate *via* lift-off. The low drying temperature and the low molecular weight of the polymer are expected to facilitate easy removal in an ultrasonic bath. An optical microscopy image in Figure 1b shows the printed high-resolution polymer pattern on the substrate before the deposition step with 2 μm gaps. After completing the process with vacuum deposition and lift-off, the resulting (ITO) pattern shown in Figure 1c has sharp features with a 2 μm linewidth.

**Figure 2:**
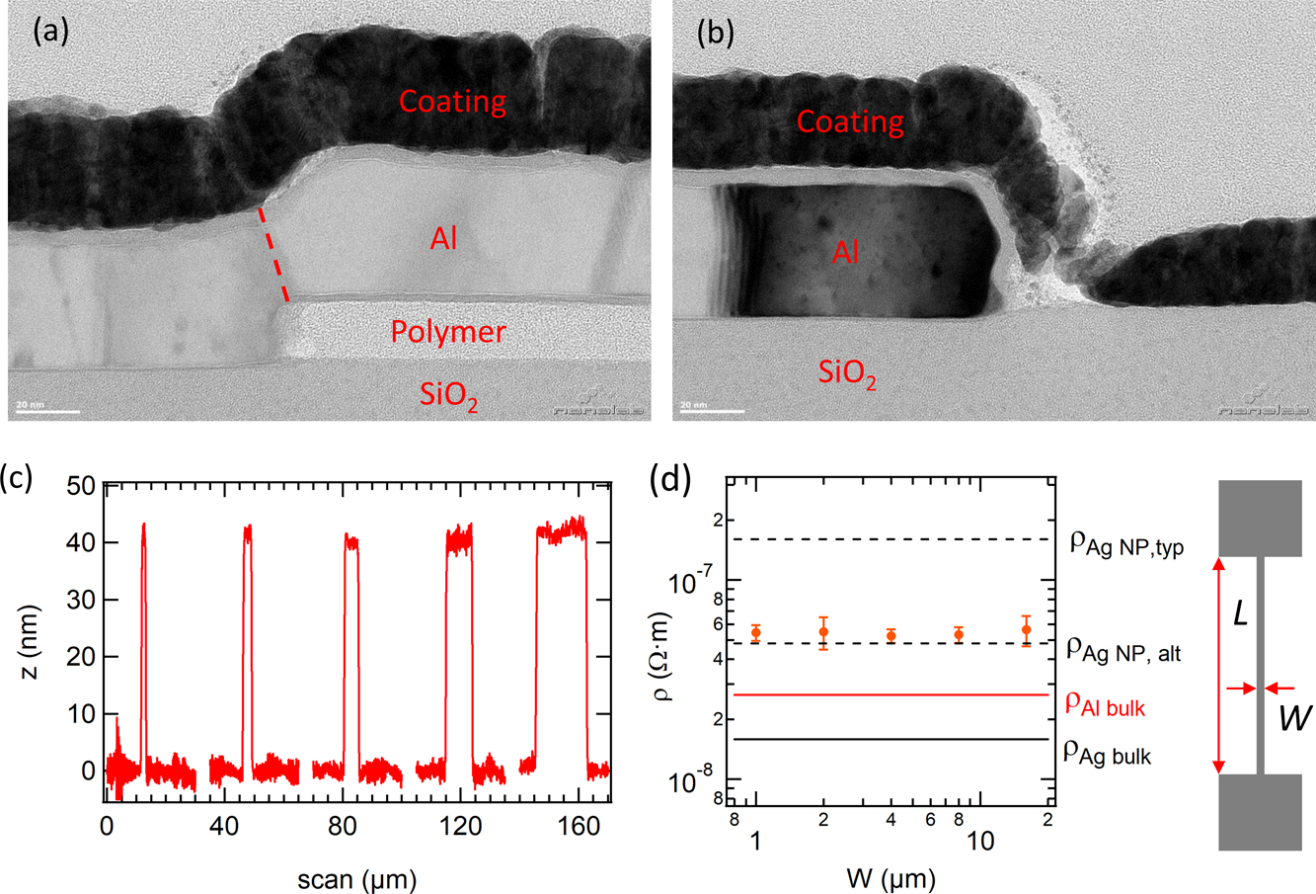
Cross-sectional TEM image (a) before and (b) after lift-off showing sharp vertical sidewalls of the ROP-processed polymer and resulting patterned Al. The weak point in the deposited film is annotated with the dashed line in panel (a). (c) Cross-sectional profiles of 1, 2, 4, 8, and 16 μm wide patterned Al-lines measured with a stylus profilometer. (d) Resistivity as a function of linewidth (*W*) for patterned ∼40 nm-thick Al-lines 500 μm in length (*L*) compared with bulk Al, bulk Ag, and printed Ag nanoparticles (NP). ρ_Ag NP, typ_ and ρ_Ag NP, alt_ denote the typical resistivity obtained with thermal sintering at temperatures compatible with low-cost plastics (<180 °C) and the lowest resistivity obtained with alternative, less-scalable low-temperature sintering methods. Error bars denote the max/min error.

To understand the details of the process, the cross-sectional profiles, the chemical composition of the ROP-processed polymer layer (step v shown in Figure 1a), and the resulting patterned layer of evaporated Al (step viii shown in Figure 1a) were studied. Transmission electron microscopy (TEM) images in Figure 2a and Figure 2b show the printed polymer layer and the deposited Al-layer before and after the lift-off, respectively. The polymer layer is uniform in thickness and has a vertical edge, which leads to a vertical edge also in the Al-layer after the lift-off. Notably, even in this sample where the lift-off was successful in only parts of the printed area due to the low thickness (∼20 nm) of the printed polymer, a thicker (∼40 nm) Al-layer was successfully patterned in the analysis region. The TEM image suggests that the Al-layer oxidizes both at the top surface and at the polymer-Al interface, where Al could readily draw oxygen from the resist layer due to its high affinity to oxygen. The oxygen-containing interface is verified in the elemental analysis in Figure S4 where oxygen is evident at the interface between the polymer and the deposited Al, both in the electron energy loss spectrum (EELS) map (Figure S4b) and the extracted line profile showing relative elemental composition (Figure S4c). Such a compound interface could result in good adhesion between the polymer and the overlaying Al film, causing the Al to be removed by exfoliation in conjunction with the polymer layer that is removed during the lift-off step. However, the oxidized interface is not solely contributing to the patterning process as the patterning method was equally applicable to other deposited materials such as SiO and ITO (*vide infra*). Less interface oxidation is expected to occur for these materials due to the weaker oxygen affinity of the constituting metal atoms (Si, In, and Sn) when compared to Al. Another potential mechanism contributing to the patterning process could be caused by PDMS oligomers transferring from the blanket surface to the top of the polymer layer,^5^ which could, in turn, lower the adhesion of the deposited material on top of the polymer resist. By comparing the Fourier transform infrared (FTIR) spectrum measured from the fabricated PDMS blanket and the ROP-processed polymer layer (Figure S4d), it is evident that no signs of PDMS oligomer transfer were detected. The spectra of the spin-coated polymer layer (processed without contact to PDMS) and the ROP-processed polymer layer are also identical. Moreover, no Si signal was detected at the polymer-Al interface in the EELS analysis.

Based on these observations, we conclude that the formation of the high-quality patterns with sharp sidewalls is likely not adhesion-related but can be attributed to the vertical sidewall profile of the ROP-processed polymer layer. The patterning of the ink occurs in the semi-dry state by the fracturing of the ink at the edges of the features on the cliché, which allows the formation of the sharp vertical sidewalls.^29^ This is in contrast to other printed lift-off methods by conventional printing methods, where the ink is patterned in the liquid state and, thus, is prone to interactions between the ink and the receiving substrate. This leads to oblique sidewalls and an uneven layer thickness. Such imperfections in the resist layer give rise to edge ears in the lift-off process as the material is deposited also on the slanted sidewalls of the resist.^30^ In this work, the vertical sidewalls of the polymer avoid similar sidewall deposition when the resist layer is thicker than the deposited layer. In addition, well-defined weak points at the top of the edges of the resist enable the patterning of deposited films with thickness close to or even higher than the resist layer thickness. As the polymer that has not been cross-linked is readily dissolved during the lift-off step, the overlaying deposited film will hang from the weak point at the edge that is annotated in Figure 2a. Ultrasonic agitation-induced cavitation can exfoliate the loosely hanging films,^31^ and repeated shear stress from the sound pressure will remove any remaining edge deposits and result in clean edges.^32^

The resist thickness can be varied both by controlling the ink coating parameters and tailoring the weight loading of the polymer in the ink. In ROP, the thickness of the ink layer is in general related to the patterning resolution such that the increasing ink thickness limits the maximum attainable linewidth resolution.^29^ This is also observed for the polymer resist ink such that ∼200–400 nm-thick polymer films could only be patterned for the large pad structures (∼100 μm level) with 5–7.5 wt % PVPh in ethyl lactate. Thicker inks will typically lead to cohesive failure of the ink during patterning in the semi-dry state. Therefore, typically, a resist thickness of ∼60–80 nm was used to pattern films down to ∼1–2 μm resolution.

The cross-sectional profile of patterned Al-lines is shown in Figure 2c with nominal widths ranging rightward from 1 to 16 μm. The thickness of the patterns is uniform regardless of the pattern width, and the sidewalls of the features are vertical, sharp, and free of any imperfections such as edge ears, thus resembling the cross-sectional profiles obtained with conventional photolithography. By measuring the resistance of the aforementioned Al-lines, the resistivity (ρ) can be calculated as a function of linewidth, as shown in Figure 2d for 500 μm long lines. The typical resistivity of printed Ag NP inks obtained using thermal sintering at temperatures compatible with low-cost plastics (at <180 °C) is in the order of 10× bulk Ag resistivity, which is shown as reference (ρ_Ag NP, typ_).^33^ The obtained ∼5 × 10^–8^ Ωm resistivity is ∼2× bulk Al (ρ_Al bulk_) and ∼3.4× bulk Ag resistivity (ρ_Ag bulk_). A similar resistivity level can be obtained for Ag NP inks at the lowest but only when alternative but less scalable low-temperature sintering methods, such as chemical, electrical, plasma, or laser sintering, are used (ρ_Ag NP, alt_).^23^ Notably, the hybrid method is a low-temperature process with mild heating at 100 °C applied only to dry the printed polymer pattern. Figure S5 shows the calculated sheet resistance (*R*_sh_) and ρ for lines with *L* = 500 and 1500 μm. The sheet resistance is on the average *R*_sh_ = 1.34 ± 0.11 Ω, which is similar to *R*_sh_ reported for high-resolution patterns formed by ROP of Ag NP ink (350 nm thick).^34^ It has been estimated that ROP has no clear disadvantage in the cost per sheet of printing of Ag nanoparticles on the polyethylene naphthalate (PEN) A4-sized substrate when compared to screen printing as, eventually, the substrate cost will dominate.^35^ However, when estimating only the material cost for a sample of 20 cm × 20 cm in size, i.e., excluding the substrate cost, the hybrid method for patterned Al is approximated to be from 1000× to 10,000× cheaper than the aforementioned ROP-processed Ag NP-based conductor, where the main cost arises from the expensive Ag NP inks (estimated with ∼10–100 $/g) (Table S5 in the Supporting Information).

**Figure 3:**
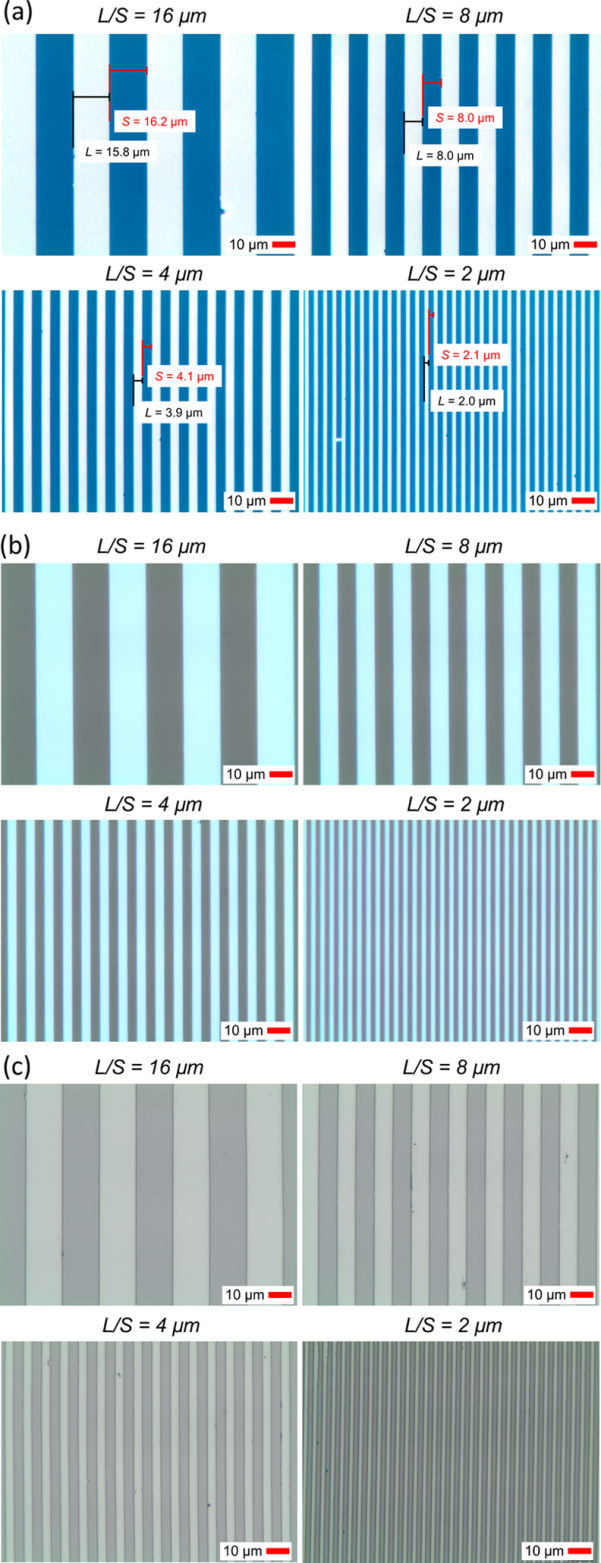
Patterned line/space patterns (L/S) for (a) evaporated ∼40 nm-thick Al, (b) sputtered ∼50 nm-thick ITO, and (c) evaporated ∼100 nm-thick SiO.

To test the applicability of the method to other materials and deposition methods, the patterning of evaporated SiO and sputtered ITO was studied. ITO sputtering was performed without intentional sample heating to avoid any damage to the polymer ink. Line/space (L/S)-patterns of Al, SiO, and ITO were tested to probe the resolution limit with L/S ranging from 16 μm down to 1 μm. Good patterning results are obtained for all materials down to L/S = 2 μm, as shown in Figure 3. For Al, L/S-patterns of 1 μm were possible; however, many samples had some missing spaces, thus indicating failed patterning by excess removal of the polymer ink. The cross-sectional profiles of the L/S-patterns shown in Figure S6 for Al and SiO indicate that no edge ears were observed in the dense L/S-patterns.

**Figure 4:**
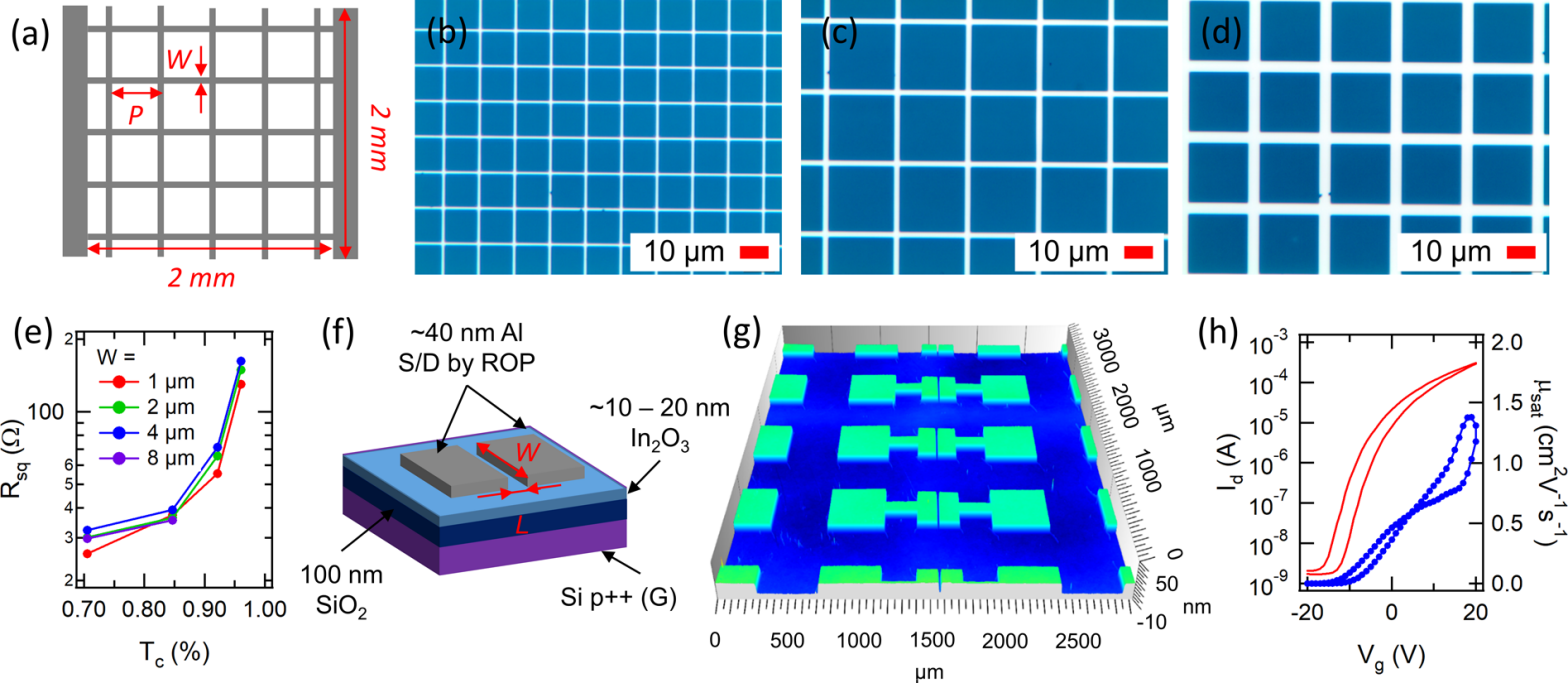
(a) Schematic image of metal mesh test patterns with varied widths (*W*) and grid periods (*P*). Optical microscope of the patterned mesh with (b) *W* = 1 μm and *P* = 6.25 μm, (c) *W* = 2 μm and *P* = 12.5 μm, and (d) *W* = 4 μm and *P* = 25 μm. (e) Measured sheet resistance (*R*_sq_) as a function of the calculated percentage of the transparent area (*T*_c_) for varied widths ranging from 1 to 8 μm. (f) Schematic image of solution-processed In_2_O_3_ thin-film transistors with Al S/D electrodes patterned by the hybrid method. (g) 3D microscopy image of TFT S/D patterns with a 10 μm channel length. (h) Transfer characteristics and calculated saturation mobility (μ_sat_) of the In_2_O_3_ TFT.

Two application examples were studied with patterned Al to demonstrate the use of the method: (i) transparent metal mesh conductors and (ii) S/D electrodes for solution-processed metal-oxide TFTs. Transparent metal mesh electrodes can be used in displays and touch panels,^36^ current collectors for photovoltaic cells,^37^ transparent cathodes for organic light-emitting diodes (OLEDs),^37^ and in transparent heaters.^38^ Metal mesh electrode test patterns (Figure 4a) with various grid linewidths (*W*) ranging from 1 to 8 μm and grid periods (*P*) ranging from 6.25 to 100 μm were patterned using ∼40 nm-thick Al. The pads at both ends of the grid pattern enable the measurement of the square resistance of the grids (*R*_sq_) of 2 × 2 mm in size. The percentage of the transparent area (*T*_c_) can be calculated as *T*_c_ = (*P* – *W*)^2^/*P*^2^ to estimate the transparency of the grids. In general, the patterning quality of the grids is high as can be seen from the optical microscopy images in Figure 4b–d, which show grids with constant *T*_c_ = 0.71 for *W* values of 1, 2, and 4 μm and *P* values of 6.25, 12.5, and 25 μm, respectively. Failure modes are observed as filled grid openings or as missing lines, but mostly occurring for the densest grids (i.e., smallest *T*_c_) (Figure S7). However, infrequent errors are not expected to impact the transparency or the square resistance as long as they are not clustered. Notably, the large-area non-uniformity of the patterning method that arises likely from the hand transfer process needs further attention in the future work. *R*_sq_ is plotted as a function of *T*_c_ as in Figure 4e for various *W* values. *R*_sq_ follows a similar increasing trend for an increase in *T*_c_ regardless of *W* due to the lowering density of conductive wires. For example, for *T*_c_ = 0.85, the square resistance is approximately 35 Ω_□_ for all *W*. Such a resistance level should be sufficient, for example, for transparent heater films in deicing applications.^38^

Solution processing and, in particular, printing of oxide TFTs are considered as viable alternative low-cost fabrication routes to sputtering in flexible display and sensor applications.^39^ However, the lack of printable low work function metals providing good Ohmic contacts to the n-type semiconductors and the high annealing temperature of printed conductive oxides are the main obstacles for achieving fully printed oxide TFTs at low temperatures that are compatible with low-cost plastic substrates (<180 °C).^40,41^ S/D contacts made from Ag NPs suffer from poor charge injection properties leading to low charge carrier mobility and charge trapping, whereas evaporated metals such as Al, Ti/Au, and Mo provide superior performance. However, these materials are difficult to form as printable NP dispersions either due to high melting points (*e.g.*, Mo) or rapid oxidation in air (Ti and Al).^8,39,42^ Here, we show that Al patterned by the hybrid method can be used as an S/D contact for solution-processed In_2_O_3_ reported earlier by our group.^43^Figure 4f shows the schematic image of the TFT, where ∼40 nm-thick Al is patterned as S/D electrodes on top of spin-coated In_2_O_3_ on an oxidized Si wafer. S/D electrodes with well-defined gaps between the electrodes are shown in Figure 4g for TFTs with a channel length of *L* = 10 μm. An example of the unoptimized TFT devices with a non-patterned semiconductor layer shows a current ON/OFF ratio >10^5^ and saturation mobility >1 cm^2^/(Vs) in the transfer curve (Figure 4h). The mobility is close to the level obtained for solution-processed In_2_O_3_ TFTs with Al S/D electrodes evaporated using a shadow mask.^40^ We expect these parameters to be improved in the future with a fully patterned semiconductor and further process optimization. Although, the effect of the patterning process on the work function of the deposited metals needs attention in the future, such effects are expected to be less important for top contact devices as demonstrated here.

The results shown here are performed using hand transfer with a rubber hand roller, where the control of the printing pressure is limited, and as expected, the large-area uniformity is not yet optimal. The large-area uniformity of the patterning is anticipated to be improved when using an ROP tool with a printing parameter control. In addition, the thickness uniformity of the polymer ink could be improved using co-solvents to control the drying of the ink, which, in turn, could improve the uniformity of the patterning process. Considering the used equipment here, the obtained micrometer-level patterning resolution is an encouraging result for the applicability of the method in printed and flexible electronics manufacturing. Besides the applications shown here, the hybrid patterning method could be applied in the fabrication of various flexible electronic devices such as sensors, antennas, resistive random access memories, passive components, and electrodes for optoelectronic components.

## Conclusions

A scalable, low-temperature, and high-resolution patterning method was employed to deliver micrometer-level structures from vacuum-deposited materials. The hybrid method combining printing and vacuum processing was based on the sequential steps of high-resolution reverse-offset printing of the polymer resist, vacuum deposition, and lift-off in an organic solvent in ultrasonic bath. The patterning of the resist ink in the semi-dry state enabled sharp, vertical sidewalls in the polymer, which allowed the patterning of the deposited material (here, Al, SiO, and ITO) using lift-off without forming edge ears. The method provided features with cross-sectional profiles resembling those obtained with photolithography with vertical sidewalls, uniform thickness regardless of the feature size, and low line edge roughness. Besides the applications shown here, namely, transparent metal mesh conductors and S/D electrodes to oxide TFTs, the method is expected to be widely applicable in printed and flexible electronics applications. The method is compatible with other printing methods and could help expand the limited material palette currently available for printed electronics to include high-performance materials (metals, semiconductors, and dielectrics) that are needed by the electronics industry.

## Methods

### Ink Synthesis and Printing

The initial screening for the polymer resist ink was performed using a table-top reverse-offset machine and flexible metal cliché with ∼1–2 μm deep patterns (described in ref (34)) on 1 min O_2_ plasma-treated (200 W, Diener Nano) 38 μm-thick polyimide (Xenomax) substrates. The polymers used were poly(methyl methacrylate) (PMMA, *M*_w_ ∼120 k Sigma-Aldrich), polyvinylpyrrolidone (*M*_w_ ∼10 k, Sigma Aldrich), and poly(4-vinylphenol) (*M*_w_ ∼22 k, Polysciences Inc., USA). The solvents were 1-butanol, 2-methoxyethanol, cyclohexanone, ethyl acetate, ethyl lactate, and toluene. The final resist inks were synthesized by dissolving either (i) poly(4-vinylphenol) in ethyl acetate or ethyl lactate in 4 wt % concentration or (ii) polyvinylpyrrolidone in 1-butanol in 5 wt % concentration. The viscosity and the surface tension of the inks and their solvents were measured using an m-VROC (Rheosense Inc., USA) small sample viscometer at a 10^4^ s^–1^ shear rate and Kibron Ez-Phi^plus^ (Kibron Inc., Finland), respectively. The ink was filtered using a 1 μm PTFE filter and coated onto an ∼ 5 cm × 5 cm sized PDMS blanket (SIM-240 and CAT-240 with a 10:1 oligomer-to-curing agent mixing ratio, Shin-Etsu) pre-treated with 6 s of O_2_ plasma using a capillary slit coater on the table-top reverse-offset machine. The contact angles of deionized water (DIW) and diiodomethane (DIM) were measured using an optical tensiometer Attension Theta Flex (Biolin Scientific, Sweden) on the PDMS blanket before and after plasma treatment to calculate the surface energy using the Fowkes theory.^44^ The ink was allowed to reach semi-dry conditions on the PDMS before patterning with a high-resolution cliché using hand transfer, i.e., applying impression through PDMS using a rubber hand roller. The patterned polymer ink was transferred onto the receiving substrate similarly by hand transfer and dried in oven at 100 °C for 10 min. The patterned, typically ∼60–80 nm thick, polymer ink resist was used as a sacrificial mask layer for evaporated Al (∼40 nm-thick layer on an oxidized Si wafer with spin-coated In_2_O_3_), evaporated SiO (∼100 nm-thick layer on borosilicate glass), or RF-sputtered ITO (∼50 nm-thick layer on borosilicate glass). For testing the method for the patterning of Al as S/D contacts for solution-processed TFTs, as metal mesh transparent conductors, and for determining the resistivity of the patterned Al lines from the same samples, 0.2 M In-nitrate hydrate (In(NO_3_)_3_*x*H_2_O, Epivalence Ltd., UK) was first dissolved in 2-methoxyethanol (details of the ink synthesis are reported in ref (43)). The ink was then filtered using a 0.2 μm PTFE filter onto oxidized 6” Si wafers with 100 nm SiO_2_ thickness after 1 min of O_2_ plasma treatment and spin-coated at 6 kRPM. The ink was dried at 130 °C for 15 min and thermally annealed on a hotplate at 300 °C for 30 min to yield an ∼10–20 nm-thick In_2_O_3_ semiconductor layer, as reported earlier in ref (43). As the resistivity of the In_2_O_3_ layer was high, the layer had negligible contribution to the metal mesh sheet resistance and Al resistivity calculations (i.e., nA-level currents in measurements with several mA values). The wafer was post-annealed at 150 °C for 30 min before TFT characterization after the evaporation and lift-off steps. The sheet resistance of the ITO grown without intentional substrate heating or post-annealing was ∼77 Ω measured from a reference Si wafer. The lift-off was performed by a 15 min ultrasonic bath (VWR USC300 THD at 45 kHz/80 W) in methanol for all samples.

### Cliché Fabrication

Cliché fabrication was carried out with a single lithography process using the standard bulk micromachining techniques. The desired patterns were transferred from a photolithographic mask to a thin layer of a positive photoresist on the silicon wafers using an i-line stepper lithography tool from Canon (FPA-3000i4). The minimum linewidth that can be reliably exposed by this tool was 0.35 μm; this guarantees the high-resolution requirement of the cliché’s fine structures down to 1 μm. After lithography, the cliché patterns were etched into the silicon with a desired depth using the photoresist as a mask. The special DRIE (deep reactive ion etching) process for the silicon etch (using Aviza Omega i2l) ensures that the critical dimensions (CD) will have the minimum deviations (<100 nm) from the mask definition or the linewidth of the photoresist.^45^ The sidewalls of the silicon structures after etching were also maintained as low as 50 nm in RMS (root mean square) roughness so that it reduces the friction with the polymer ink during the ink patterning processes.

### Characterization

The printing and the patterning results were characterized using an optical microscope, 3D microscope in coherence scanning interferometry mode (Sensofar S Neox), and stylus profilometer (Dektak 150). ITO layer thickness was measured from a reference Si wafer using a dual-angle ellipsometer (Filmtek 4000). All electrical characterizations were performed using a Keithley 4200 SCS semiconductor analyzer in the dark, except for the ITO sheet resistance, which was measured with a four-point probe. TEM images before and after lift-off were taken using a JEOL JEM-2100 with a LaB_6_ source at 200 kV in bright field mode. The lamellae for TEM were prepared using the FIB lift-out technique and capped with carbon-based spin-on-glass and Pt prior to milling.

## Supporting Information Available

List of selected solvents for ROP polymer ink; results and microscopy images of initial screening for suitable polymer resist ink for ROP; identified ROP resist inks and their viscosity and surface tension; contact angle goniometry results on oxygen plasma-treated PDMS and calculated surface energies; characterization of the high-resolution clichés; sheet resistance and conductivity of patterned Al-lines for various lengths; STEM/EELS analysis of samples before lift-off as the elemental map and line profile; FTIR analysis of PDMS blanket, spin-coated, and ROP-processed polymer resist layers; 3D microscope and stylus profilometer cross-sectional profile for various Al and SiO L/S-patterns; optical microscopy images and corresponding sheet resistance for various Al metal mesh patterns; and estimation of the cost of materials for conductors fabricated by the hybrid method and by ROP of Ag NPs (PDF)
