# Supplementary material for: Reverse-Offset Printing of Polymer Resist Ink for Micrometer-Level Patterning of Metal and Metal-Oxide Layers
Source: ACS Appl Mater Interfaces. 2021 Aug 25;13(35):41782–90. doi: 10.1021/acsami.1c08126 (PMC8431341; doi:10.1021/acsami.1c08126)
Supplement: Supplementary file 1 — am1c08126_si_001.pdf [file am1c08126_si_001.pdf]

Supporting Information:

Reverse-Offset Printing of Polymer Resist Ink for Micrometer-Level Patterning of Metal and Metal-Oxide Layers

*Asko Sneck, Henri Ailas, Feng Gao, Jaakko Leppäniemi**

VTT Technical Research Centre of Finland, Ltd., Tietotie 3, FI-02150 Espoo, Finland

*jaakko.leppaniemi@vtt.fi

Table S1. List of selected of solvents and their relevant physical properties. The PDMS absorption is estimated from silicone O-ring compatibility charts as suggested in ref. [1]. * = measured.

Parameter	1-butanol[2]	2-methoxy-ethanol[3]	Cyclo-hexanone[4]	Ethyl acetate[5]	Ethyl lactate[6]	Toluene[7]
PDMS absorption	Moderate	High	High	Moderate	Moderate	High
T_b (°C)	118	125	156	77	154	111
P_{vap} (mmHg)	~7	~6	~5	~73	~4	~28
γ (mN/m)	24.6*	31	35	23.9*	30.2*	~28
η (mPa·s)	4.0*	~1.5	2.2	~0.5*	~3.4*	~0.6

Table S2. Results of the initial screening for suitable polymer resist ink for ROP. Polymer weight percent shown in parenthesis. * = patterning depends on processing time due to fast drying

Polymer (M_w)	1-butanol	2-methoxy-ethanol	Cyclo-hexanone	Ethyl acetate	Ethyl lactate	Toluene
PMMA (120 k)	-	Precipitates (3 wt%)	Poor (3 wt%)	.	No transfer (1 - 5 wt%)	Fast drying (3 wt%)
PVP (10 k)	Good (3-5 wt%)	-	Poor (3 wt%)	Fast drying (3 wt%)	-	Fast drying (3 wt%)
PVPh (22 k)	-	OK (3 wt%)	OK (3 wt%)	Good* (3 - 4 wt%)	Good (3 wt%)	Fast drying (3 wt%)

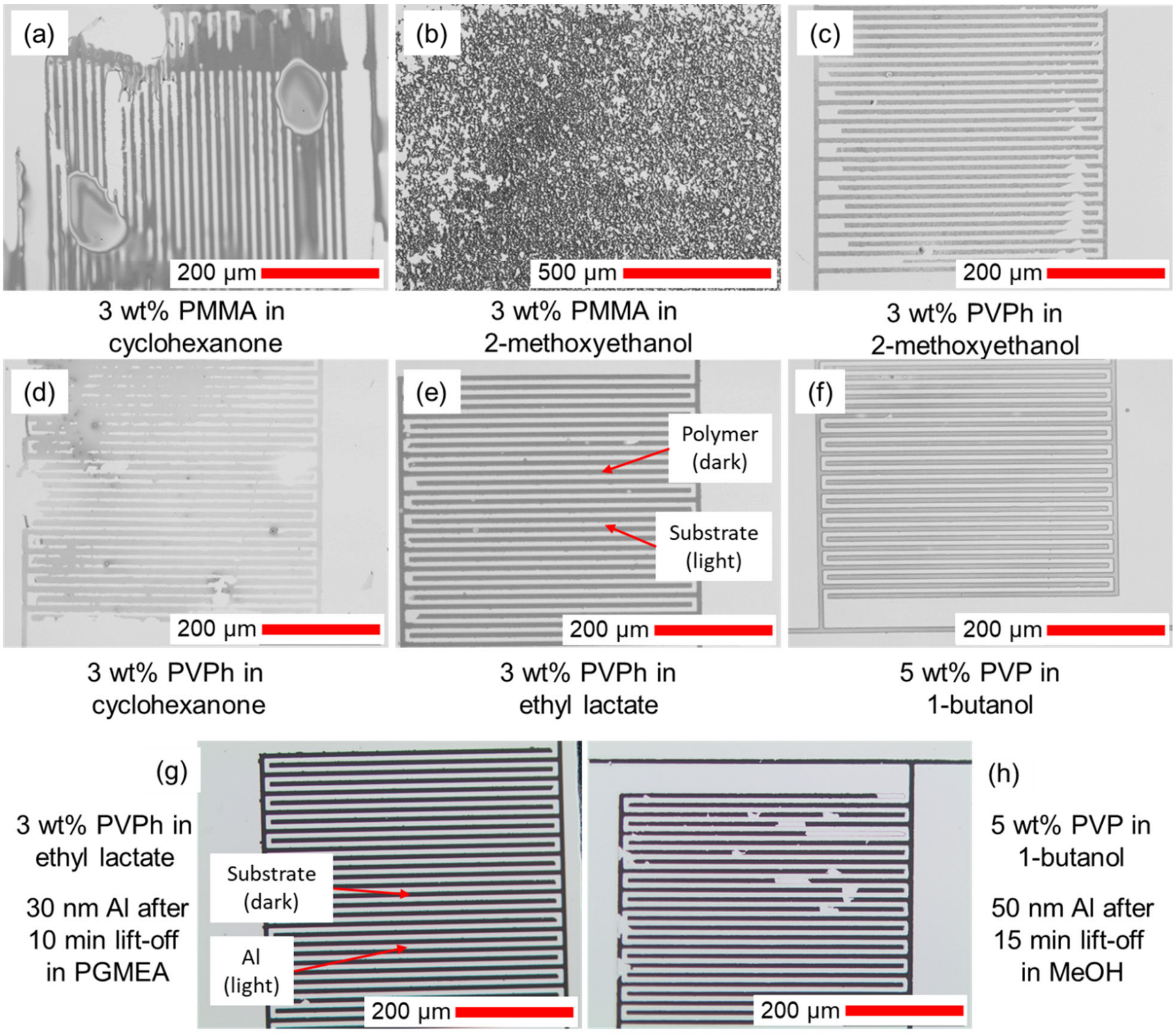

Figure S1. Optical microscope images of samples from initial ink screening tests with (a) 3 wt% PMMA in cyclohexanone, (b) 3 wt% PMMA in 2-methoxyethanol, (c) 3 wt% PVPh in 2-methoxyethanol, (d) 3 wt% PVPh in cyclohexanone, (e) 3 wt% PVPh in ethyl lactate and (f) 5 wt% PVP in 1-butanol. The nominal L/S of the test pattern is 8 µm. Test patterns after lift-off for (g) 3 wt% PVPh in ethyl lactate with 30 nm evaporated Al sonicated for 10 min in propylene glycol methyl ether acetate (PGMEA) and (h) 5 wt% PVP in 1-butanol with 50 nm evaporated Al sonicated for 15 min in methanol (MeOH).

Table S3. ROP resist inks identified suitable for the hybrid patterning process. PGMEA = propylene glycol methyl ether acetate, MeOH = methanol.

**Polymer (M_w)**	**wt% and solvent**	**γ (mN/m)**	**η (mPa·s)**	**Drying after print**	**Solvent for lift-off**	**Notes**
PVPh (~22 k)	4 wt% in ethyl acet.	23.8	0.75	10 min @ 100 °C	MeOH/ PGMEA	Ink dries fast
PVPh (~22 k)	4 wt% in ethyl lactate	29.9	5.4	10 min @ 100 °C	MeOH/ PGMEA	Ink dries slowly
PVP (~10 k)	5 wt% in 1-butanol	24.6	5.5	10 min @ 120 °C	MeOH	-

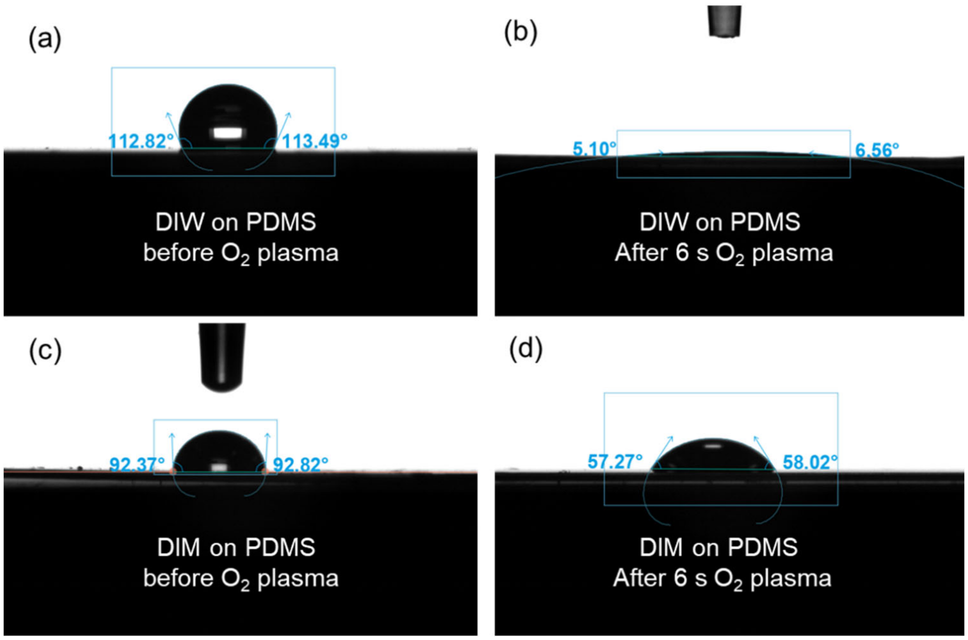

Figure S2. Contact angle goniometer images θ of deionized water (DIW) on PDMS before (a) and after (b) 6 s O_2 plasma and contact angle of diiodomethane (DIM) on PDMS before (c) and after (d) 6 s O_2 plasma.

Table S4. Average contact angle for deionized water (θ_{DIW}) and diiodomethane (θ_{DIM}) test liquids and the calculated polar (σ_p), dispersive (σ_d) and total (σ_{total}) surface energies of PDMS before and after 6 s O_2 plasma treatment.

**PDMS condition**	θ_{DIW} (°)	θ_{DIM} (°)	σ_p (mN/m)	σ_d (mN/m)	σ_{total} (mN/m)
No plasma	111.9	92.4	0.93	11.66	12.59
6 s O_2 plasma	5.1	58.7	44.02	29.31	73.33

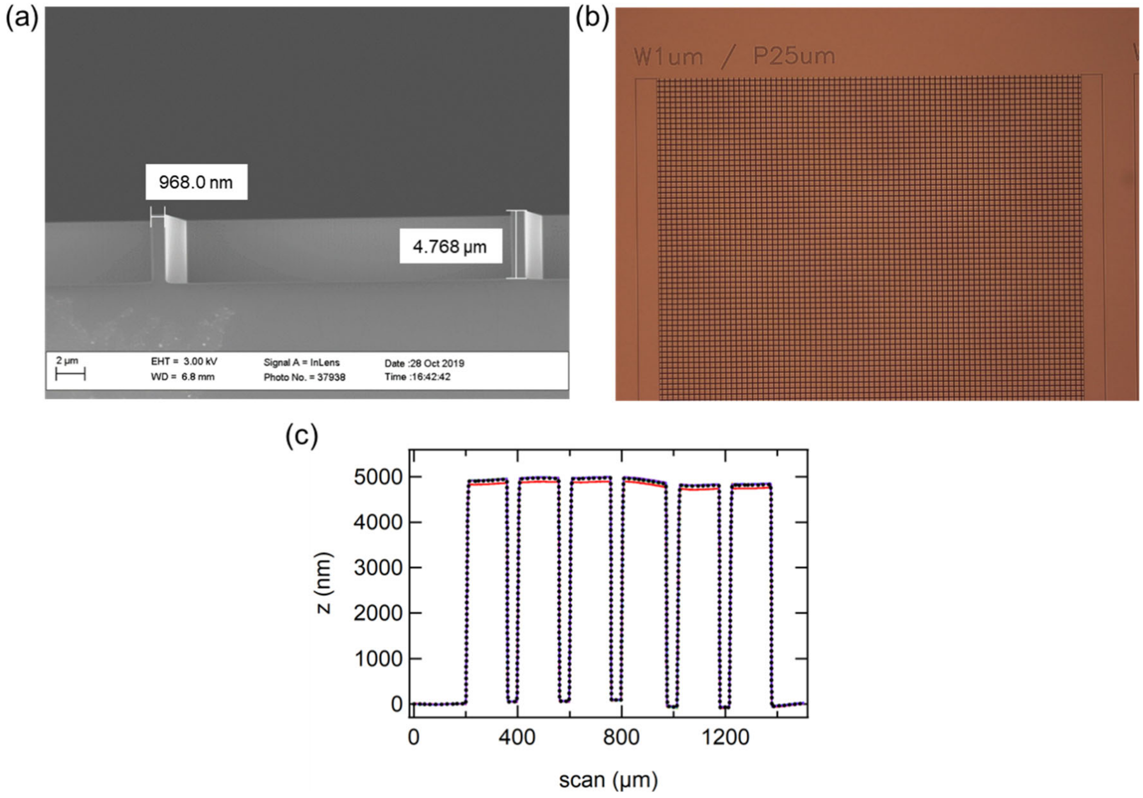

Figure S3. (a) Cross-section SEM image of high-resolution Si cliché. (b) Optical microscope image of transparent metal mesh pattern on the cliché. (c) Stylus profilometer scan over patterns in the cliché showing ~5 µm pattern depth.

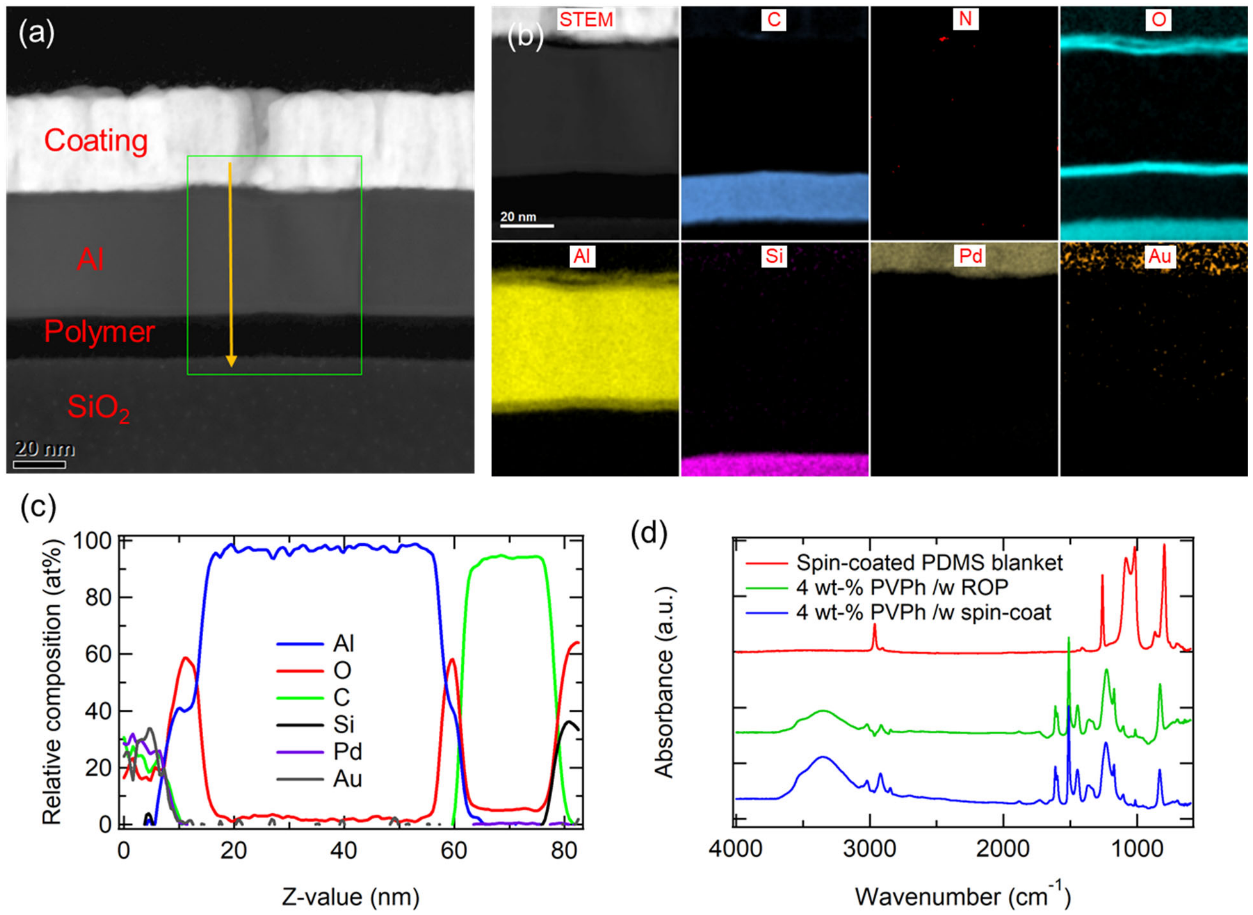

Figure S4. (a) Bright field STEM image of analyzed sample with EELS analysis area (green box) and linescan location (yellow arrow). (b) EELS analysis showing elemental maps for C, N, O, Al, Si, Pd and Au with 4 Å step size. (c) Extracted EELS linescan profile for atomic percentage of Al, O, C, Si, Pd and Au. (d) ATR FTIR spectra for spin-coated PDMS blanket, polymer resist layer (4 wt% poly(4-vinylphenol) in ethyl lactate) spin-coated (processed without contact to PDMS) and reverse-offset printed (ROP) (in contact with PDMS).

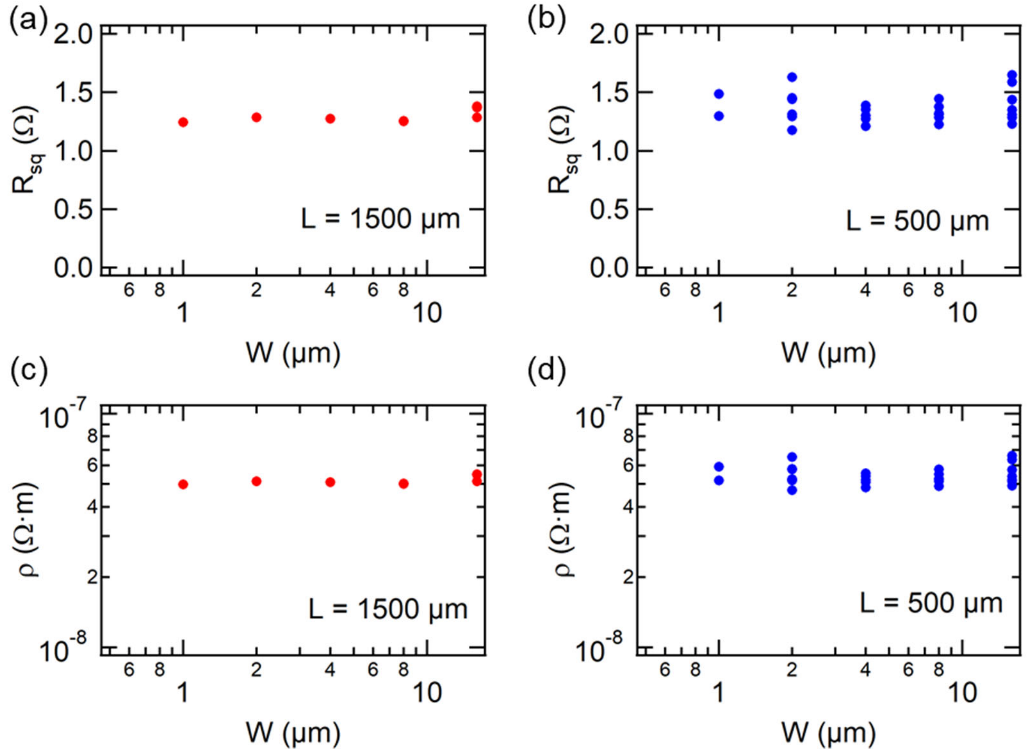

Figure S5. Square resistance as a function of linewidth for (a) 1500 μm and (b) 500 μm long Al lines with ~40 nm thickness. Calculated resistivity for (c) 1500 μm and (d) 500 μm long lines.

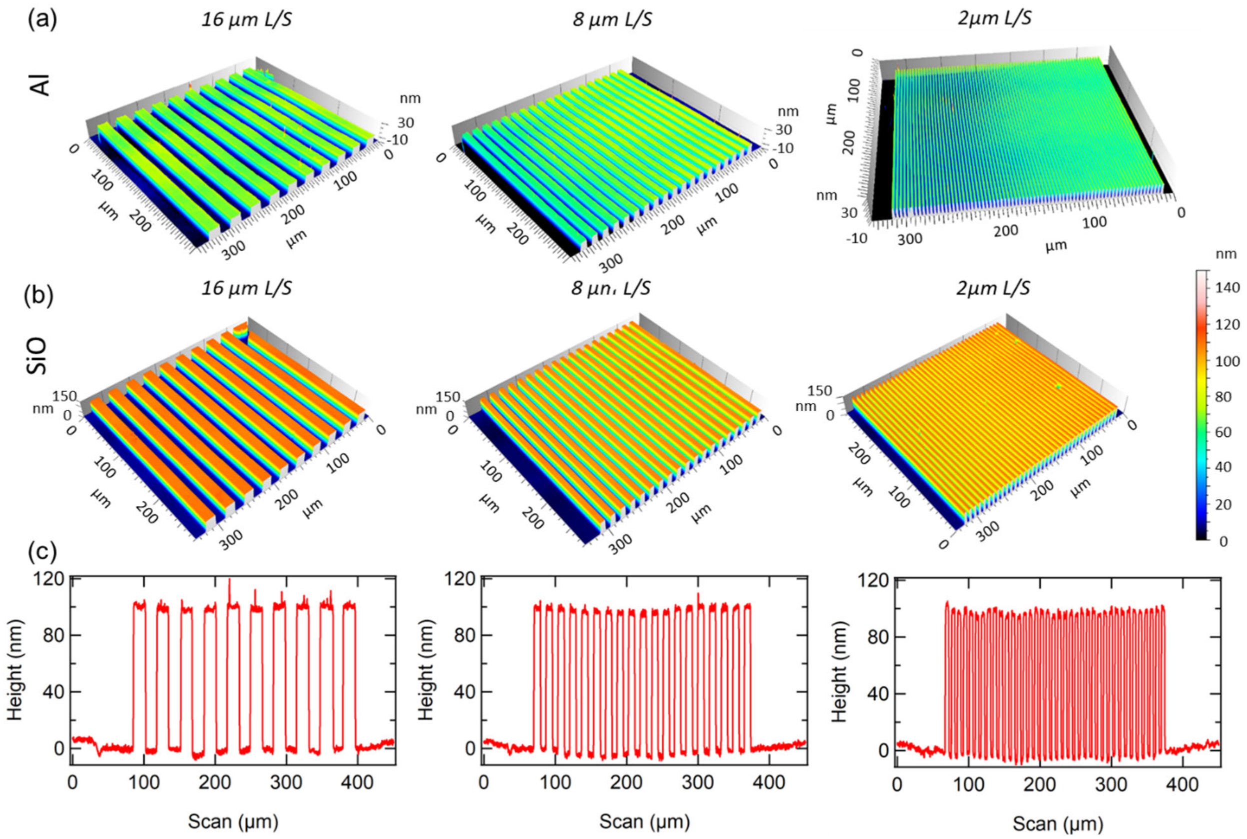

Figure S6. 3D microscope images of line/space-patterns (L/S) of 16 µm, 8 µm and 2 µm for Al patterns on glass substrate taken with coherence scanning interferometry (CSI, Sensofar Neox) for (a) Al and (b) SiO. The Z-axis data is re-scaled with the thickness measured with stylus profilometry (~40 nm for Al and ~100 nm for SiO). (c) Cross-sectional profile of the L/S-patterns shown in (b) for SiO measured with the stylus profilometer.

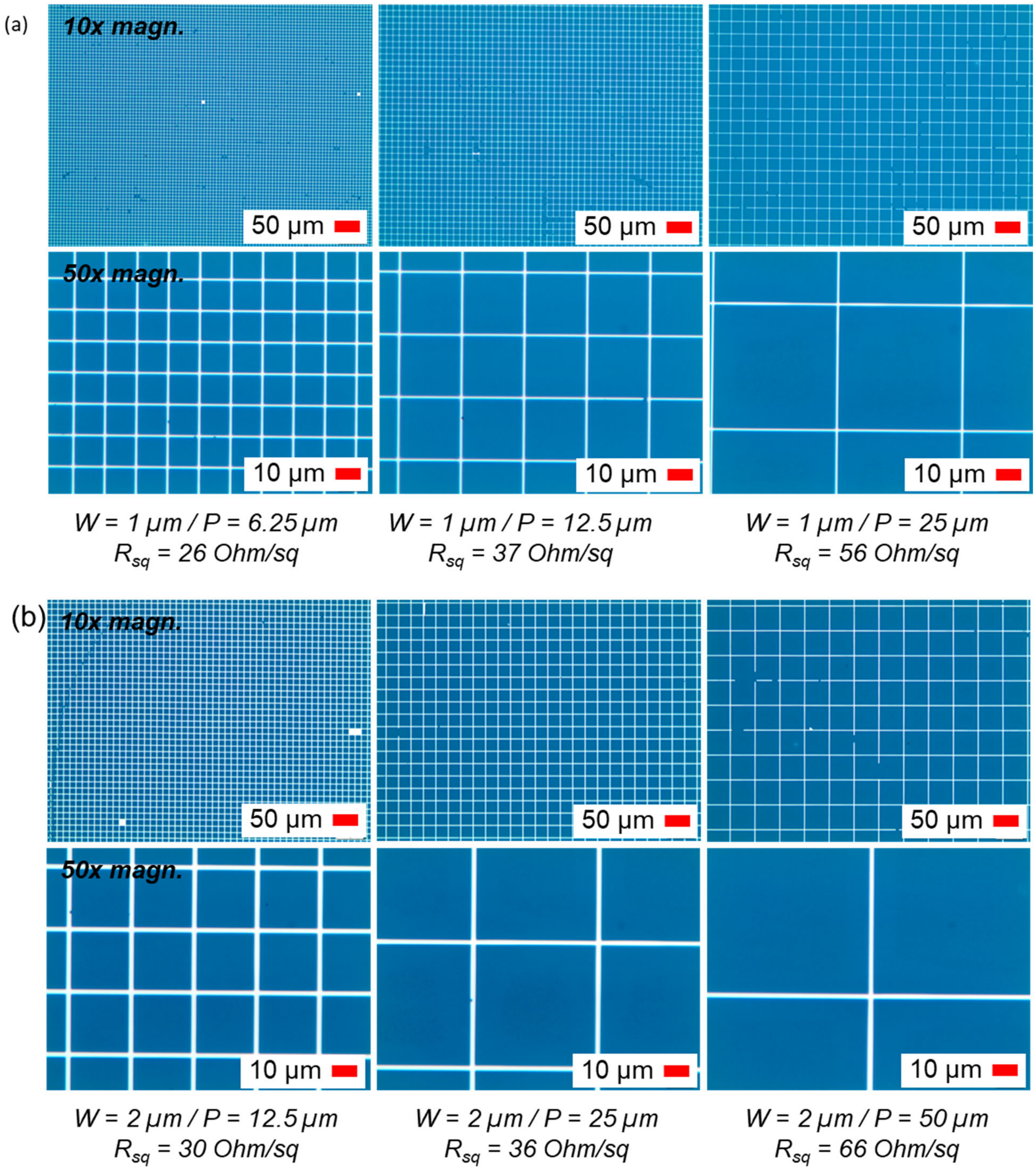
(a)
10x magn.
50 μm
50 μm
50 μm
50x magn.
10 μm
10 μm
10 μm
W = 1 μm / P = 6.25 μm
R_{sq} = 26 Ohm/sq
W = 1 μm / P = 12.5 μm
R_{sq} = 37 Ohm/sq
W = 1 μm / P = 25 μm
R_{sq} = 56 Ohm/sq
(b)
10x magn.
50 μm
50 μm
50 μm
50x magn.
10 μm
10 μm
10 μm
W = 2 μm / P = 12.5 μm
R_{sq} = 30 Ohm/sq
W = 2 μm / P = 25 μm
R_{sq} = 36 Ohm/sq
W = 2 μm / P = 50 μm
R_{sq} = 66 Ohm/sq

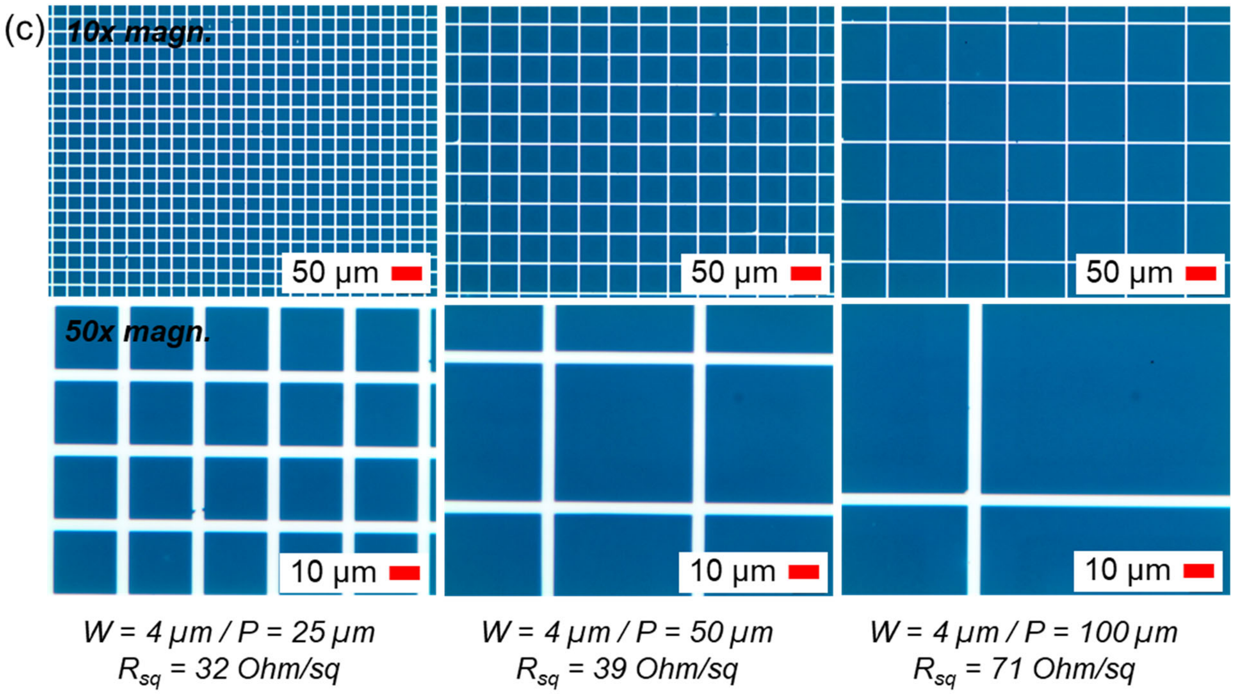

Figure S7. Optical microscope images of transparent metal mesh conductors (~40 nm thick Al) patterned with the hybrid method. Linewidth (W), grid period (P) and corresponding measured square resistance R_{sq} shown for (a) W = 1 µm, (b) W = 2 µm and (c) W = 4 µm patterns at two microscope objective magnifications (10x and 50x).

Materials cost comparison between ROP Ag NPs and the hybrid patterning method (with Al)

The cost estimation for the evaporated Al material is estimated based on the total evaporated mass from a simple point source,[9] which overestimates the material consumption

$$m_e = \frac{dm_s}{dA_s}\frac{4\pi r^2}{\cos\theta},$$

where dm_s is the material evaporated on the substrate unit area dA_s on a surface of sphere with a source-to-sample distance r. θ is the angle between the normal of the substrate and the vector from the source through the center of the unit area. In case, where the source is at $r = 40$ cm distance and the sample is 20 x 20 cm in size, $\cos\theta \approx 1$ and the equation can be approximated as

$$m_e \approx \frac{4\pi r^2 \rho_s}{d_s},$$

where ρ_s and d_s are the density and thickness of the evaporated material layer.

Table S5. Material cost calculations for Al conductor patterned using the hybrid method and conductor obtained by ROP of Ag NPs for 20 cm x 20 cm sample size delivering ~1.4 Ω_{sq} square resistance. The data for ROP of Ag NPs is from ref. [8]. * = The Ag NP ink price is an estimation. The actual price varies with the type of ink and vendor.

**Method**	**Al via hybrid method**		**Ag NPs via ROP**	
**Parameters**	**Value**	**Description**	**Value**	**Description**
Ink loading	4 wt%	Polymer wt%	-	-
d_p	100 nm	Polymer thickness	**-**	**-**
d_s	40 nm	Al thickness	350 nm[8]	Ag NP thickness
ρ_s (density)	2.7 g/cm^3	100 % bulk Al	3.15 g/cm^3	30 % bulk Ag
r	40 cm	Source distance	-	-
**Cost of materials**	**Cost**	**Description**	**Cost**	**Description**
$C_{material}$	0.176 ¢/g	Al-metal price	~10 – 100 $/g	Ag ink price*
C_{resist}	662 ¢/g	PVPh (25 k) price	-	-
$C_{solvent}$	0.04 ¢/g	Ethyl lactate price	-	-
**Calculated cost**	**Cost**	**Description**	**Cost**	**Description**
Resist layer	0.004 ¢	Cost of resist layer	-	-
Deposited layer	0.038 ¢	Cost of Al-layer	44 – 440 ¢	Cost of Ag layer
**Total cost**	**~0.04 ¢**		**~44 - 440 ¢**	

REFERENCES:

(1) Kusaka, Y.; Fukuda, N.; Ushijima, H. Recent Advances in Reverse Offset Printing: An Emerging Process for High-Resolution Printed Electronics. *Jpn. J. Appl. Phys.* **2020**, *59* (SG). https://doi.org/10.7567/1347-4065/ab6462.

(2) National Center for Biotechnology Information (2021). PubChem Compound Summary for CID 263, 1-Butanol. Retrieved June 2, 2021 from https://pubchem.ncbi.nlm.nih.gov/compound/1-Butanol.

(3) National Center for Biotechnology Information (2021). PubChem Compound Summary for CID 8019, 2-Methoxyethanol. Retrieved June 2, 2021 from https://pubchem.ncbi.nlm.nih.gov/compound/2-Methoxyethanol.

(4) National Center for Biotechnology Information (2021). PubChem Compound Summary for CID 7967, Cyclohexanone. Retrieved June 2, 2021 from https://pubchem.ncbi.nlm.nih.gov/compound/Cyclohexanone.

(5) National Center for Biotechnology Information (2021). PubChem Compound Summary for CID 8857, Ethyl acetate. Retrieved June 2, 2021 from https://pubchem.ncbi.nlm.nih.gov/compound/Ethyl-acetate.

(6) National Center for Biotechnology Information (2021). PubChem Compound Summary for CID 7344, Ethyl lactate. Retrieved June 2, 2021 from https://pubchem.ncbi.nlm.nih.gov/compound/Ethyl-lactate.

(7) National Center for Biotechnology Information (2021). PubChem Compound Summary for CID 1140, Toluene. Retrieved June 2, 2021 from https://pubchem.ncbi.nlm.nih.gov/compound/Toluene.

(8) Sneck, A.; Mäkelä, T.; Alastalo, A. Reverse-Offset for Roll-to-Roll High-Resolution Printing. *Flex. Print. Electron.* **2018**, *3*, 014001. https://doi.org/https://doi.org/10.1088/2058-8585/aa9f00.

(9) Ohring, M. *Materials Science of Thin Films*; Academic Press, 1992.
